# First real-world study on the effectiveness and tolerability of rimegepant for acute migraine therapy in Chinese patients

**DOI:** 10.1186/s10194-024-01873-5

**Published:** 2024-09-27

**Authors:** Zhao Yang, Xiaodan Wang, Mengyue Niu, Qiao Wei, Huizhu Zhong, Xiaoyan Li, Weihong Yuan, Wenli Xu, Shuo Zhu, Shengyuan Yu, Jun Liu, Jianzhou Yan, Wenyan Kang, Peijian Huang

**Affiliations:** 1 Department of Neurology, Institute of Neurology, Ruijin Hospital, Shanghai Jiao Tong University School of Medicine, No 197, Ruijin Second Road, Huangpu District, Shanghai, 20025 China; 2 Department of Neurology, Hainan Branch, Ruijin Hospital, Shanghai Jiao Tong University School of Medicine, No 41, Kangxiang Road, Qionghai, 517434 China; 3 Hainan Lecheng Institute of Real World Study, Qionghai, China; 4 Department of Neurology, Hainan General Hospital, Hainan Affiliated Hospital of Hainan Medical University, No 19, Xiuhua Road, Xiuying District, Haikou, 570311 China; 5 Department of Neurology, Chinese PLA General Hospital, Beijing, China; 6 School of International Pharmaceutical Business, China Pharmaceutical University, Nanjing, China; 7 The Research Center of National Drug Policy and Ecosystem, China Pharmaceutical University, Nanjing, China

**Keywords:** Rimegepant, Migraine, Real-world, Effectiveness, Tolerability

## Abstract

**Background:**

Rimegepant, a small molecule calcitonin gene-related peptide (CGRP) receptor antagonist, is indicated for acute and preventive migraine treatment in the United States and other countries. However, there is a lack of prospective real-world evidence for the use of rimegepant in Chinese migraine patients.

**Methods:**

This was a single-arm, prospective, real-world study. While taking rimegepant to treat migraine attacks as needed, eligible participants were asked to record their pain intensity, functional ability, and accompanying symptoms for a single attack at predose and 0.5, 1, 2, 24, and 48 h postdose via a digital platform. Adverse events (AEs) during the rimegepant treatment period were recorded and analysed. The percentages of participants who experienced moderate to severe pain at predose and 0.5, 1, 2, 24, and 48 h postdose were assessed. Additionally, the percentages of participants who reported better/good outcomes in terms of pain intensity, functional ability, and accompanying symptoms at 0.5, 1, 2, 24, and 48 h postdose were analysed. In addition, the total cohort (full population, FP) was stratified into a prior nonresponder (PNR) group to observe the effectiveness and safety of rimegepant for relatively refractory migraine and a rimegepant and eptinezumab (RE) group to observe the effectiveness and safety of the combination of these drugs.

**Results:**

By November 24th, 2023, 133 participants (FP, *n* = 133; PNR group, *n* = 40; RE group, *n* = 28) were enrolled, and 99 participants (FP, *n* = 99; PNR group, *n* = 30; RE group, *n* = 23) were included in the analysis. Rimegepant was effective in treating migraine in the FP and both subgroups, with a significant decreasing trend in the percentages of participants experiencing moderate to severe pain postdose (*p* < 0.05) and a marked increase in the percentages of participants who reported better/good outcomes in terms of pain intensity, functional ability, and accompanying symptoms at 0.5, 1, 2, 24, and 48 h postdose compared with predose. AEs were reported by 6% of participants in the FP, and all AEs were mild.

**Conclusions:**

In the real world, rimegepant is effective in the acute treatment of migraine patients in China. The low incidence rate of AEs highlighted the favourable tolerability profile of rimegepant.

**Trial registration:**

Clinicaltrials.gov NCT05709106. Retrospectively registered on 2023-02-01.

**Graphical Abstract:**

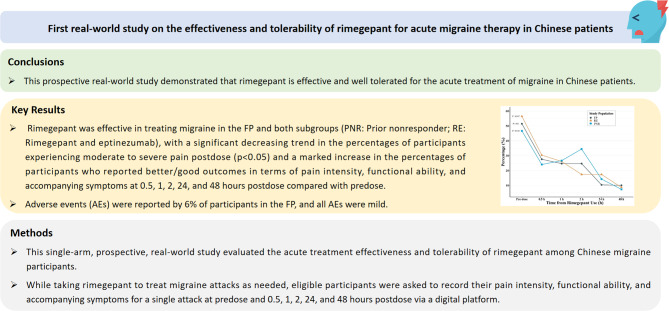

**Supplementary Information:**

The online version contains supplementary material available at 10.1186/s10194-024-01873-5.

## Background

Migraine, a chronic neurological disorder affecting approximately 151.6 million individuals in China, is characterized by moderate to severe headaches and reversible associated symptoms, such as photophobia, phonophobia, and nausea [1–3]. Globally, migraine is the second most disabling disorder across all age groups [4, 5].

In China, a variety of drugs are available for the treatment of migraine. Common medications include caffeinated analgesic compounds, nonsteroidal anti-inflammatory drugs (NSAIDs), triptans, ergotamines, etc [6, 7]. However, these conventional therapies have limitations [8]. For example, NSAIDs and triptans are contraindicated in patients with cardiovascular disease and should be used with caution in those with cardiovascular risk factors [9]. Additionally, the use of caffeinated analgesic compounds, NSAIDs, triptans, and ergotamines is associated with a risk of medication overuse headache (MOH), which is a significant concern in China [10]. Moreover, approximately one-third of patients do not respond adequately to triptans, which have long been the gold standard in migraine treatment [11]. Therefore, an effective and safe acute treatment for migraine is clearly needed.

Rimegepant, a small molecule calcitonin gene-related peptide (CGRP) receptor antagonist, is approved for the acute treatment and prevention of migraine in the United States, European Union, and United Kingdom [12]. As of the time of this data analysis, rimegepant had not been approved for use in the mainland of China. However, owing to favourable government policies in pilot zones, innovative medicines and devices that are already in use in the United States, but not yet in the mainland of China, can be accessed in hospitals in the Boao pilot zone (Hainan Province, China) after local health authority approval and a hospital application process [13]. This allows all eligible Chinese migraine patients, as identified by doctors, to use this innovative medicine in the Boao pilot zone. Among all gepants and CGRP monoclonal antibodies (mAbs), only rimegepant and eptinezumab are available in our hospital.

Pivotal and long-term safety studies conducted in the United States initially demonstrated the safety, tolerability, and efficacy of rimegepant for the acute and preventive treatment of migraine [14–18]. In addition, a phase III randomized, placebo-controlled trial (RCT) demonstrated the efficacy and safety of rimegepant orally disintegrating tablet (ODT) for the acute treatment of migraine in adults living in China or South Korea (NCT04574362) [19]. In this trial, a subsequent post hoc subgroup analysis specifically performed in Chinese adults also revealed that rimegepant was superior to placebo in terms of all primary and key secondary efficacy endpoints, with comparable safety and tolerability [3]. While RCTs have tested drugs under ideal conditions, excluding patients with more than 8 moderate to severe attacks per month, patients with more than 15 headache days per month within the last 3 months, or patients prescribed mAbs within the last 6 months, real-world studies exploring drug effectiveness and tolerability in unselected patients under routine circumstances are needed [14–16, 19]. Currently, however, there is a lack of such prospective real-world evidence for rimegepant.

Studies in China have reported that more than 40% of patients respond inadequately to acute treatment [20]. In some patients, treatment failure occurs after repeated use of conventional analgesics, and these patients progress to resistant or refractory migraines [21]. A post hoc analysis of three phase III treatment trials demonstrated that rimegepant was effective in the acute treatment of migraine in adults with a history of insufficient response to one or more triptans [22]. To our knowledge, no real-world studies have specifically demonstrated the efficacy and safety of rimegepant in migraine patients with a history of nonresponse to other analgesics.

Furthermore, in clinical studies and in practice, rimegepant is also often used to treat breakthrough migraine in patients receiving preventive treatment [6]. A long-term safety study reported that approximately 14% (*n* = 243) of patients were taking other medications for migraine prevention during long-term rimegepant treatment [23]. Among these patients, 13 were taking an anti-CGRP mAb as prophylaxis and received rimegepant for acute treatment [23]. However, there are limited data demonstrating the efficacy and safety of the combined use of rimegepant and anti-CGRP mAbs. A case study of two patients receiving erenumab monthly showed promising preliminary efficacy and safety of its combination with rimegepant [24]. Given that the CGRP pathway is a target for both acute and preventive treatments and that these treatments are sometimes used together in the same patient, there is a need for more data on the acute treatment effects of the coadministration of rimegepant and anti-CGRP mAbs in real-world clinical practice [25, 26].

Therefore, the objective of this study was to evaluate the real-world effectiveness and tolerability of rimegepant for the acute treatment of migraine. Furthermore, we explored the effectiveness and tolerability of rimegepant in migraine patients with a history of nonresponse to other analgesics or with combined use of rimegepant and eptinezumab.

## Methods

### Study setting

This prospective study was conducted at the Neurology Clinic of Ruijin-Hainan Hospital affiliated with Shanghai Jiaotong University School of Medicine. The study was conducted in accordance with the Declaration of Helsinki and Good Clinical Practice (GCP) guidelines and was approved by the Institutional Ethics Committees of our hospital. All participants signed an informed consent form (ICF) before undergoing any study procedures. The study was registered at ClinicalTrials.gov (number NCT05709106).

## Study population

Adults who were diagnosed with migraine (with or without aura) according to the International Classification of Headache Disorders, 3rd Edition (ICHD-3) [2] and were prescribed rimegepant as part of routine treatment determined by the prescribing physician were screened for this study. The exclusion criteria were individuals who were unable to complete the questionnaire and follow-up, pregnant and lactating females, individuals who were highly dependent on medical care, and individuals with any other conditions deemed unsuitable for study participation by the investigator. Patients who met none of the exclusion criteria, were not participating in any other clinical trials and signed an ICF were enrolled consecutively.

## Study design and procedures

This was a prospective, single-arm, real-world study with the latest data collected on November 24th, 2023. At the baseline visit, all eligible participants completed the Chinese versions of two scales: the Migraine-Specific Quality-of-Life Questionnaire (MSQ) version 2.1 and the Headache Impact Test-6 (HIT-6) [27, 28]. Demographic information and medical history were also collected.

During the study period, participants took rimegepant ODT (75 mg) to treat migraine attacks as needed. They recorded pain intensity on a four-point scale (0 = none, 1 = mild, 2 = moderate, 3 = severe), functional ability on a four-point scale (0 = normal function, 1 = mild impairment, 2 = severe impairment, 3 = required bedrest), and accompanying symptoms on a binary scale (nausea, photophobia, phonophobia, etc.; 0 = absent, 1 = present) for a single attack at predose (the time right immediately taking rimegepant) and 0.5, 1, 2, 24, and 48 h postdose. Any analgesics, including additional doses of rimegepant, taken within 48 h after the initial dose of rimegepant were also recorded. The participants were required to report adverse events (AEs) during the rimegepant treatment period. All the information was collected via a digital platform, which automatically sent weekly reminders to the participants until they completed and submitted the questionnaire evaluating the effectiveness of rimegepant treatment on their mobile devices.

The total cohort (full population, FP) was stratified into a prior nonresponder (PNR) group and a rimegepant and eptinezumab (RE) group. PNRs were defined as participants who reported a nonresponse to any acute or preventive medications for migraine treatment in the month before enrolment. RE use was defined as the use of rimegepant to treat migraine attacks with the use of eptinezumab to prevent migraine attacks. The participants received 100 mg of eptinezumab as an intravenous infusion over approximately 30 min every 3 months. Effectiveness and tolerability were evaluated in all three groups.

## Endpoints

### Effectiveness

Effectiveness was measured as the percentage of participants experiencing moderate to severe pain at predose and 0.5, 1, 2, 24, and 48 h postdose. The percentage of participants who reported better/good outcomes in terms of pain intensity, functional ability and accompanying symptoms at 0.5, 1, 2, 24 and 48 h postdose compared with predose was also measured to determine relative change. A better/good pain intensity outcome was defined as any decrease in pain intensity compared with the predose level or no change in pain intensity but only mild pain or pain freedom. A better/good functional ability outcome was defined as any improvement in functional ability compared with the predose level or no change in functional ability but only mild impairment or normal function. A better/good accompanying symptoms outcome was defined as any decrease in the number of accompanying symptoms compared with the predose number or the absence of accompanying symptoms.

### Tolerability

The tolerability of rimegepant was assessed in terms of the AEs reported by the participants during the rimegepant treatment period.

### Statistical analysis

Participants who took rimegepant for the acute treatment of a migraine attack and provided data at predose and at least 3 postdose time points were included in the analysis. Baseline information and effectiveness and safety data for the three groups (FP, PNR subgroup and RE subgroup) were analysed.

We first summarized demographics and baseline characteristics. Continuous data with a normal distribution are expressed as the means and standard deviations (SDs), whereas medians and interquartile ranges (IQRs) are reported for continuous variables with a skewed distribution. Categorical variables are expressed as frequencies and percentages. For the percentages of participants who reported better/good outcomes in terms of pain intensity, functional ability, and accompanying symptoms at different time points postdose compared with predose in the three groups, 95% confidence intervals (CIs) were calculated. Generalized estimating equations (GEEs) were used to examine the trends in postdose pain intensity in the FP, PNR and RE groups. Safety was assessed in participants who took rimegepant for the acute treatment of migraine in all three groups. To explore the effectiveness of acute treatment with rimegepant in patients with very high frequency migraine (VHFM) days, we additionally analysed the effectiveness endpoints in participants with more than 15 monthly migraine days (MMDs).

SAS software, version 9.4 (SAS Institute Inc., Cary, NC, USA) and R version 4.3.2 (package: ggplot2 for plots) were used for the analyses, and *p* < 0.05 was considered statistically significant.

## Results

### Participants

A total of 133 participants (FP) were enrolled in this study up to November 24th, 2023, 40 (30.1%) of whom were allocated to the PNR subgroup and 28 (21.1%) of whom were allocated to the RE subgroup. Participants who took rimegepant for the acute treatment of migraine, those who satisfactorily completed the corresponding questionnaires and those who provided data at predose and at least 3 postdose time points were eligible for the analysis. Overall, 99 participants were included in the analysis, with 30 participants in the PNR subgroup and 23 participants in the RE subgroup.

### Demographic and baseline characteristics

The patient demographic and baseline characteristics are presented in Table 1. For the FP, the majority of patients were female (72.7%), with a mean (SD) age of 41.4 (10.4) years. The mean (SD) age at migraine onset was 19.8 (9.1) years. Most participants (77.8%) had 3 or more accompanying symptoms during migraine attacks. In the month before the baseline visit, the median number of MMDs was 10 (IQR: 5.0–20.0), and 35.4% of the participants were prescribed preventive treatment.

**Table 1:** Demographic and baseline characteristics

Demographic	FP(*n* = 99)	PNR(*n* = 30)	RE(*n* = 23)
Age, mean (SD), y	41.4 (10.4)	41.5 (9.8)	42.2 (10.3)
Female, n (%)	72 (72.7)	24 (80.0)	16 (69.6)
BMI, mean (SD), kg/m^2^	22.7 (3.5)	22.9 (3.1)	23.5 (3.8)
Age at migraine onset, mean (SD), y	19.8 (9.1)	18.9 (8.9)	19.7 (9.5)
Number of accompanying symptoms, n (%)			
0	2 (2.0)	0 (0.0)	0 (0.0)
1	7 (7.1)	2 (6.7)	1 (4.4)
2	13 (13.1)	2 (6.7)	2 (8.7)
≥ 3	77 (77.8)	26 (86.7)	20 (87.0)
Accompanying symptoms, n (%)			
Dizziness	39 (39.4)	13 (43.3)	11 (47.8)
Nausea	79 (79.8)	26 (86.7)	19 (82.6)
Vomiting	65 (65.7)	25 (83.3)	19 (82.6)
Phonophobia	74 (74.7)	24 (80.0)	20 (87.0)
Photophobia	69 (69.7)	20 (66.7)	16 (69.6)
Others	50 (50.5)	14 (46.7)	10 (43.5)
Family history of migraine, n (%)	61 (61.6)	17 (56.7)	14 (60.9)
Menstrual-related migraine, n (%)^a^	51 (70.8)	14 (58.3)	12 (75.0)
Primary migraine type, n (%)			
Migraine without aura	53 (53.5)	18 (60.0)	16 (69.6)
Migraine with aura	43 (43.4)	11 (36.7)	7 (30.4)
Unknown^b^	39 (3.0)	1 (3.3)	0 (0.0)
MMDs in the past month, median (IQR), d	10.0 (5.0–20.0)	11.0 (6.5–25.0)	15.0 (7.5–20.5)
MMDs frequency, n (%)			
0–3 d	16/99 (16.2)	3/30 (10.0)	0/23 (0.0)
4–7 d	21/99 (21.2)	5/30 (16.6)	6/23 (26.1)
8–14 d	21/99 (21.2)	8/30 (26.7)	3/23 (13.0)
≥ 15 d	41/99 (41.4)	14/30 (46.7)	14/23 (60.9)
Drug treatments in past month, n (%)			
Participants on acute treatment	81 (81.8)	26 (86.7)	18 (78.3)
Participants on preventive treatment	35 (35.4)	19 (63.3)	10 (43.5)
Participants on acute and preventive treatments	24 (24.2)	15 (50.0)	7 (30.4)
AEs in the past month, n (%)	42 (42.4)	16 (53.3)	13 (56.5)
Nonresponse in the past month, n (%)			
Nonresponse to any acute treatment	20 (20.2)	20 (66.7)	4 (17.4)
Nonresponse to any preventive treatment	16 (16.2)	16 (53.3)	5 (21.7)
Nonresponse to any acute or preventive treatment	30 (30.3)	30 (100.0)	7 (30.4)
HIT-6 score, mean (SD)^c^	65.6 (6.7)	68.8 (5.3)	65.5 (6.4)
Severe impact (60–78), n (%)^c^	84 (87.5)	28 (96.6)	18 (81.8)
Substantial impact (56–59), n (%) ^c^	6 (6.2)	1 (3.45)	3 (13.6)
Some impact (50–55), n (%)^c^	4 (4.2)	0 (0.0)	1 (4.5)
Little or no impact (36–49), n (%)^c^	2 (2.1)	0 (0.0)	0 (0.0)
MSQ score, mean (SD)^c^			
Role restrictive	45.0 (20.5)	35.6 (20.5)	44.8 (21.3)
Role preventive	51.1 (22.0)	42.0 (22.4)	50.4 (22.6)
Emotional function	57.5 (26.6)	43.6 (27.2)	58.0 (26.1)

Abbreviations No, number; SD, standard deviation; y, years; BMI, body mass index; MMDs, monthly migraine days; NSAIDs, nonsteroidal anti-inflammatory drugs; AEs, adverse events; HIT-6, Headache Impact Test-6; FP, full population; PNR, prior nonresponder; RE, rimegepant and eptinezumab

^a^ Measured only in women

^b^ Participants reported that they were not sure about their aura symptoms during previous migraine attacks

^c^ Participants number = 96 for the FP, 29 for the PNR subgroup and 22 for the RE subgroup

The participants in the PNR subgroup had demographic and baseline characteristics that were similar to those of the participants in the FP, except the PNR subgroup had a greater number of participants with 3 or more accompanying symptoms, a greater incidence of vomiting (83.3% vs. 65.7%), greater use of preventive treatment in the past month (63.3% vs. 35.4%) and a greater percentage of participants with severe headache-related disability (96.6% vs. 87.5%).

Compared with the FP, the RE subgroup had a greater incidence of vomiting (82.6% vs. 65.7%) and phonophobia (87.0% vs. 74.7%), a greater median number of MMDs (15.0 days vs. 10.0 days), a greater incidence of very high-frequency (≥ 15 days) MMDs (60.9% vs. 41.4%) and a higher rate of preventive treatment use (43.5% vs. 35.4%) in the month before the baseline visit.

### Acute treatment effectiveness

**Fig. 1:**
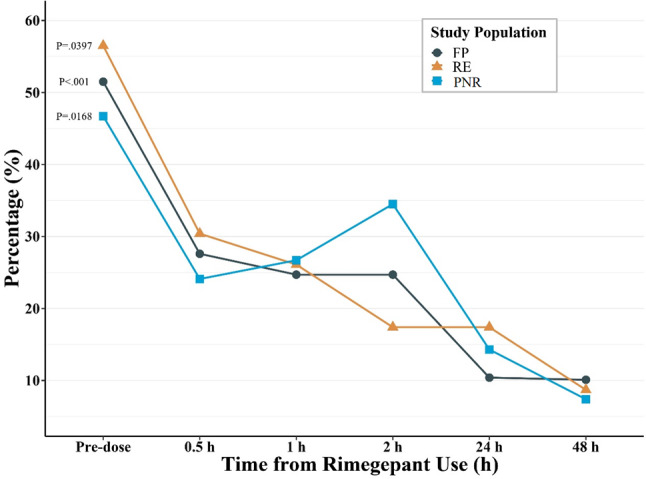
Percentage of participants who experienced moderate to severe pain at different time points in the study population. Abbreviations FP, full population; PNR, prior nonresponder; RE, rimegepant and eptinezumab

Rimegepant showed a significant trend in decreasing the percentages of participants who experienced moderate to severe pain in all three groups (*p* < 0.05) (Fig. 1). The percentage of participants who experienced moderate to severe pain decreased from 51.5% at predose to 10.1% at 48 h postdose, from 46.7% at predose to 7.41% at 48 h postdose, and from 56.5% at predose to 8.7% at 48 h postdose in the FP, PNR and RE groups, respectively (Supplementary Table 1). Compared with predose levels, rimegepant significantly increased the percentages of participants who reported better/good outcomes in terms of pain intensity, functional ability, and accompanying symptoms at 0.5, 1, 2, 24 and 48 h postdose in the FP and the two subgroups (Table 2). Sixteen (16.2%) participants in the FP, 3 (10.0%) participants in the PNR subgroup and 7 (30.4%) participants in the RE subgroup used other rescue analgesics, predominantly NSAIDs, caffeinated analgesic compounds and acetaminophen, within 48 h postdose. None of the participants used additional rimegepant doses within 48 h. Additionally, 3 (3.0%) participants in the FP, 2 (6.7%) in the PNR subgroup and 1 (4.3%) in the RE subgroup were also on preventive therapy (preventive treatment other than eptinezumab) during the migraine attack.

**Table 2:** Participants who reported better/good outcomes in terms of pain intensity, functional ability and accompanying symptoms

Numbers of participants^a^	FP		PNR		RE	
	99		30		23	
**Better/good outcomes in terms of pain intensity**	**n/N (%)**	**95% CI**	**n/N (%)**	**95% CI**	**n/N (%)**	**95% CI**
0.5 h postdose	73/98 (74.5)	(65.9, 83.1)	22/29 (75.9)	(60.3, 91.4)	16/23 (69.6)	(50.8, 88.4)
1 h postdose	76/97 (78.4)	(70.2, 86.5)	24/30 (80.0)	(65.7, 94.3)	18/23 (78.3)	(61.4, 95.1)
2 h postdose	75/97 (77.3)	(69.0, 85.7)	21/29 (72.4)	(56.1, 88.7)	20/23 (87.0)	(73.2, 100.7)
24 h postdose	87/96 (90.6)	(84.8, 96.5)	25/28 (89.3)	(77.8, 100.7)	19/23 (82.6)	(67.1, 98.1)
48 h postdose	81/89 (91.0)	(85.1, 97.0)	25/27 (92.6)	(82.7, 102.5)	21/23 (91.3)	(79.8, 102.8)
**Better/good outcomes in terms of functional ability**	**n/N (%)**	**95 %** ***CI***	**n/N (%)**	**95 %** ***CI***	**n/N (%)**	**95 %** ***CI***
0.5 h postdose	88/98 (89.8)	(83.8, 95.8)	24/29 (82.8)	(69,0, 96.5)	22/23 (95.7)	(87.3, 104.0)
1 h postdose	87/97 (89.7)	(83.6, 95.7)	26/30 (86.7)	(74.5, 98.8)	22/23 (95.7)	(87.3, 104.0)
2 h postdose	84/97 (86.6)	(79.8, 93.4)	22/29 (75.9)	(60.3, 91.4)	22/23 (95.7)	(87.3, 104.0)
24 h postdose	92/96 (95.8)	(91.8, 99.8)	26/28 (92.9)	(83.3, 102.4)	21/23 (91.3)	(79.8, 102.8)
48 h postdose	88/89 (98.9)	(96.7, 101.1)	26/27 (96.3)	(89.2, 103.4)	22/23 (95.7)	(87.3, 104.0)
**Better/good outcomes in terms of accompanying symptoms**	**n/N (%)**	**95 %** ***CI***	**n/N (%)**	**95 %** ***CI***	**n/N (%)**	**95 %** ***CI***
0.5 h postdose	64/97 (66.0)	(56.6, 75.4)	20/29 (69.0)	(52.1, 85.8)	15/23 (65.2)	(45.8, 84.7)
1 h postdose	76/96 (79.2)	(71.0, 87.3)	23/30 (76.7)	(61.5, 91.8)	16/23 (69.6)	(50.8, 88.4)
2 h postdose	74/94 (78.7)	(70.4, 87.0)	22/29 (75.9)	(60.3, 91.4)	17/23 (73.9)	(56.0, 91.9)
24 h postdose	88/95 (92.6)	(87.4, 97.9)	26/28 (92.9)	(83.3, 102.4)	19/23 (82.6)	(67.1, 98.1)
48 h postdose	82/88 (93.2)	(87.9, 98.4)	26/27 (96.3)	(89.2, 103.4)	22/23 (95.7)	(87.3, 104.0)

Abbreviations n, number of people who reported better/good outcomes in terms of pain intensity, functional ability or accompanying symptoms; N, number of participants; FP, full population; PNR, prior nonresponder; RE, rimegepant and eptinezumab

^a^ Number of participants included in the analysis

We also explored the effectiveness of acute rimegepant treatment in participants with VHFM days (≥ 15 MMDs), and rimegepant also showed a significant trend in decreasing the percentages of participants who experienced moderate to severe pain (*p* < 0.05) (Supplementary Fig. 1). More detailed results can be found in Appendix 2.

### Safety and tolerability

Only a small number of participants experienced AEs after taking rimegepant. The overall number of AEs was 6 (6.1%) in the FP, 4 (13.3%) in the PNR subgroup and 1 (4.3%) in the RE subgroup (Table 3). All reported AEs were mild. The most frequently reported AEs (those reported in ≥ 1.0% of the rimegepant-treated participants) in the FP were abdominal bloating (*n* = 2, 2.02%), stomach bloating (*n* = 2, 2.02%), gastric discomfort (*n* = 1, 1.01%), nausea (*n* = 1, 1.01%), insomnia (*n* = 1, 1.01%), drowsiness (*n* = 1, 1.01%), salivation (*n* = 1, 1.01%) and dizziness (*n* = 1, 1.01%).

**Table 3:** Percentage of participants who reported adverse events (AEs) in the study population

AEs, *n*(%)	FP (*n* = 99)	PNR (*n* = 30)	RE (*n* = 23)
Any AEs	6(6.1)	4(13.3)	1(4.3)
Most common AEs			
Abdominal bloating	2(2.0)	2(6.7)	0
Stomach bloating	2(2.0)	2(6.7)	0
Salivation	1(1.0)	1(3.3)	1(4.3)
Drowsiness	1(1.0)	1(3.3)	0
Gastric discomfort	1(1.0)	1(3.3)	0
Dizziness	1(1.0)	1(3.3)	0
Nausea	1(1.0)	0	0
Insomnia	1(1.0)	0	0

Abbreviations No, number; FP, full population; PNR, prior nonresponder; RE, rimegepant and eptinezuma

## Discussion

This study provides real-world data on the efficacy and safety of rimegepant in the acute treatment of migraine. Compared with the general migraine cohort, the study population exhibited greater disease severity, characterized by a prolonged duration of migraine (21.6 years vs. 10.2 years), a higher frequency of attacks (≥ 15 MMDs; 41.4% vs. 20.9%), a greater number of participants with menstrual-related migraine (70.8% vs. 9.80%) [29], and a greater number of participants on preventive treatment (35.4% vs. 16.5%) [30]. Additionally, the quality of life and functional ability of many participants, as evaluated by the MSQ and HIT-6, were severely impacted. This may be attributed to the fact that rimegepant was only available in the Boao pilot zone in China at the time of this data analysis, necessitating additional time and money for patients to travel to obtain the medication. Consequently, more patients with severe disease may have been inclined to visit Boao. The approval of rimegepant in the mainland of China in January 2025 is expected to provide easier access for more patients [31].

To our knowledge, greater disease severity might be associated with more difficult treatment. However, the results demonstrated that rimegepant was effective and well tolerated in the acute treatment of migraine in Chinese adults. Similarly, the results in the PNR subgroup indicated that rimegepant was effective in patients with a history of prior nonresponse to other acute or preventive analgesics. Furthermore, in the RE group, the results revealed that rimegepant was effective in treating breakthrough migraines in patients treated with eptinezumab, particularly those with VHFM days or chronic migraine. Although the majority of participants who came to our hospital had greater disease severity, there were still a small number of participants with mild conditions who had fewer MMDs or experienced little or no impact on function (Table 1). Therefore, these findings can be generalized to migraine patients with different disease severities. However, these data must be interpreted with caution when extrapolating these findings to patients outside China, as this study was only conducted in Chinese migraine patients.

Specifically, rimegepant showed a significant trend in decreasing the percentages of participants who experienced moderate to severe pain in all three groups (*p* < 0.05). The effects were all very prominent as early as 0.5 h postdose and were sustained for up to 48 h, confirming both early onset and durable effects of this acute migraine treatment. The slight increase in the trend of moderate to severe pain from 0.5 to 2 h postdose in the PNR subgroup could be attributed to the greater disease severity in some participants and the relatively small sample size, where changes in three participants accounted for almost a 10% increase. However, the overall results in the PNR subgroup also revealed a significant decreasing trend in the percentage of participants who experienced moderate to severe pain (*p* < 0.05). Additionally, rimegepant significantly improved pain, function and accompanying symptoms by increasing the percentage of participants who reported better/good outcomes in terms of pain intensity, functional ability and accompanying symptoms at 0.5, 1, 2, 24 and 48 h postdose in all three groups. At 48 h postdose, all the parameters improved to greater than 90%.

The results in the PNR group were consistent with those of a post hoc analysis of three phase III treatment trials, which revealed that rimegepant was effective for acute migraine treatment in adults with a history of insufficient response to 1 or ≥ 2 triptans [22]. Although the limited number of participants in this group restricted a detailed analysis of the effectiveness and tolerability of rimegepant among those with a history of nonresponse to specific analgesics, our study suggested that rimegepant can be an effective option for patients in China with insufficient response or nonresponse to other analgesics. The limited number of participants in the PNR subgroup was due primarily to the study design, which included only participants who reported nonresponse to any analgesics in the month before the baseline visit. Participants who reported nonresponse to other analgesics more than a month prior to enrolment were not included in the PNR subgroup. However, according to the information provided by the participants during hospital visits, most had tried almost all available analgesics in the mainland of China but could not achieve good migraine control. These patients were more inclined to spend additional time and money to visit the pilot zone and use innovative medicines that are not yet approved for use in the mainland of China. Therefore, a more comprehensive treatment history might reveal a greater number of participants with a history of treatment failure.

In the RE subgroup, the results were generally consistent with those of a previous study, which demonstrated that the combination of a CGRP receptor antagonist with an anti-CGRP mAb was safe and efficacious [24, 32]. These findings were further supported by the results of a case study conducted with two patients receiving monthly erenumab, in which all 16 attacks were alleviated by rimegepant, allowing them to discontinue other migraine medications [24]. Additionally, neither of the two patients experienced AEs with rimegepant alone nor in combination with erenumab [23, 24]. Eptinezumab is a newly developed monoclonal antibody that targets CGRP for the prevention of migraine and acts on the same pathway as rimegepant. It has been suggested that mAbs and small-molecule agents might act synergistically by targeting different parts of the CGRP pathway or enhancing the blocking effect [33, 34]. Therefore, rimegepant is effective and safe for treating breakthrough migraine in patients receiving preventive treatment.

Sixteen (16.2%) participants in the FP, 3 (10.0%) participants in the PNR subgroup and 7 (30.4%) participants in the RE subgroup used other rescue analgesics within 48 h postdose, with slightly higher percentages than those in phase III studies of rimegepant [19]. The main reason behind this is that we extended the collection time of the rescue medication from 24 h to 48 h in our study compared with phase III studies [14–16, 19], which would inevitably increase the proportion of participants who used rescue medications in the 24–48 h period. In addition, this was also mainly due to the greater disease severity in this real-world study. For example, participants in this study had a longer migraine history than those in the phase III study did (21.6 years vs. 10.0 years) and a higher frequency of attacks [19]. In the phase III study, the inclusion criteria were participants with two to eight migraine attacks with moderate or severe pain intensity per month and < 15 days per month with migraine or nonmigraine headache [19]. However, the participants with ≥ 15 MMDs accounted for 41.4% of the FP, 46.7% of the PNR subgroup and 60.9% of the RE subgroup in this study. Additionally, a greater number of participants would have had a history of treatment nonresponse than that reported in our study. The participants usually came to our hospital to try rimegepant because they had no other choices in the mainland of China. Another important reason may be that many participants stopped using preventive medication after initiating rimegepant treatment. At baseline, 35.4% of the participants in the FP, 63.3% in the PNR subgroup and 43.5% in the RE subgroup were on preventive treatment, whereas only 3 (3.0%) participants in the FP, 2 (6.7%) in the PNR subgroup and 1 (4.3%) in the RE subgroup (preventive treatment other than eptinezumab) were on prevention therapy during the tracked rimegepant-treated attack. An important aim of prevention treatment is to increase the response rate to acute therapy [35]. Therefore, the large number of participants in this study who stopped preventive therapy may have influenced the treatment response to rimegepant. The reason why so many participants stopped preventive treatment may be the effect of rimegepant on MMD reduction, even when used as needed [36, 37], which we also found in our study and will further analyse in a subsequent study. Additionally, the small sample size may also have contributed to this higher percentage, as a small number of participants with very severe migraine were taking as many as 11 kinds of analgesics at baseline. For these participants, we suggested gradually reducing the number of analgesics. With respect to the specific analgesics taken, most participants used NSAIDs, caffeinated analgesic compounds and acetaminophen, which is consistent with a survey conducted in China [38].

Approximately 71% of the female participants in this study reported experiencing menstrual-related migraines. Menstrual migraine attacks have consistently been reported to be more disabling, less responsive to symptomatic treatment, longer in duration, and more prone to relapse than nonmenstrual migraine attacks [39]. While a subgroup analysis specifically targeting menstrual-related migraine patients was not conducted, the overall results of the FP at least demonstrate in part that rimegepant was effective and well tolerated for the treatment of menstrual-related migraine. A limitation of this study was the absence of data regarding whether the migraine attacks treated with rimegepant were menstrual-related. More data are needed in the future to confirm these findings.

The incidence of AEs reported in the FP in this study was very low (6.1%), even lower than that reported in phase III studies, which also demonstrated the favourable safety profile of rimegepant [19]. Additionally, all the AEs were mild. These real-world findings confirm that rimegepant is safe and well tolerated for the treatment of migraine.

### Strengths and limitations

Our study has several strengths. Compared with phase III studies, this study provides important information on treatment effectiveness and safety of rimegepant in a “real-world” population. And the data from this study contribute additional insights on the real-world effectiveness of rimegepant in patients with a history of nonresponse, patients concurrently using eptinezumab, and patients with VHFM days. In addition, this real-world study allowed patients to treat their migraine attacks at mild pain intensity or at the very onset of migraine, which may help patients get a better outcome. As early administration of acute care medication could be important in suppressing the progression of the sensitization in the trigeminovascular pathway and preventing the progression of the migraine attack to include central sensitization [44]. Furthermore, the study design involved automated reminders and a digital platform used on cell phones to administer the relevant questionnaires, which significantly increased the convenience and compliance of the participants and increased the completeness and accuracy of the data.

Nevertheless, our study has several limitations. The main limitation of this study lies in the inherent limitations of observational studies. This includes the lack of a randomization process. As the objective of this real-world study was to evaluate participants receiving rimegepant treatment for migraine attacks, this analysis also did not include a control group. In addition, we could not determine the specific timing when the patients took the medication to treat a migraine attack. As the timing of medication use may influence drug effectiveness [35, 40–44], we did not use the endpoint of pain freedom at 2 h postdose, which is usually the primary endpoint of phase III studies. In addition, we cannot ensure that the results reported here were based on the first dose of rimegepant or other doses. The response to the first dose may differ slightly from responses to multiple doses, as shown in other study [41]. To address this concern, we encouraged the participants to record the data as early as possible, and the digital platform automatically sent reminders to the participants weekly until they submitted the questionnaire to reduce differences. Therefore, the results of this study can still demonstrate the real-world effectiveness of rimegepant and provide a reference and directions for future studies. Another potential limitation was that this was a single-centre study. However, due to the pilot zone policy, the participants enrolled in this study were from across China. The third limitation is that the data were self-reported by the participants, resulting in some recall bias. To improve the accuracy of the data, the study personnel checked the data in a timely manner and asked participants whether they had any questions.

## Conclusions

This study demonstrated that rimegepant is effective and well tolerated for the acute treatment of migraine in Chinese patients, even those with VHFM days, a history of nonresponse, and concomitant eptinezumab use. Overall, these results suggest that rimegepant is a promising treatment option for Chinese migraine patients.

## Electronic supplementary material

Below is the link to the electronic supplementary material.

Supplementary Material 1

## Data Availability

The datasets used and/or analysed during the current study are available from the corresponding author on reasonable request.

## Abbreviations

AEs: Adverse events
CGRP: Calcitonin gene-related peptide
FP: Full population
GCP: Good Clinical Practice
GEEs: Generalized estimating equations
HIT-6: Headache Impact Test-6
ICF: Informed consent form
ICHD-3: International Classification of Headache Disorders, 3rd Edition
IQR: Interquartile range
MOH: Medication overuse headache
mAbs: Monoclonal antibodies
MSQ: Migraine-Specific Quality-of-Life Questionnaire
MMDs: Monthly migraine days
NSAIDs: Nonsteroidal anti-inflammatory drugs
ODT: Orally disintegrating tablet
PNR: Prior nonresponder
RCT: Randomized, placebo-controlled trial
RE: Rimegepant and eptinezumab
SD: Standard deviation
VHFM: Very high frequency migraine
