# Supplementary material for: First real-world study on the effectiveness and tolerability of rimegepant for acute migraine therapy in Chinese patients
Source: J Headache Pain. 2024 Sep 27;25(1):160. doi: 10.1186/s10194-024-01873-5 (PMC11438109; doi:10.1186/s10194-024-01873-5)
Supplement: Supplementary file 1 — Supplementary Material 1 [file 10194_2024_1873_MOESM1_ESM.docx]

**Appendix 1 The percentage of participants experiencing moderate to severe pain in the FP, PNR subgroup and RE subgroup**

**Supplementary Table 1. The percentages of participants who experienced moderate to severe pain from predose to 48 hours postdose**

|  | | **FP** | | | **PNR** | | | **RE** | |
| --- | --- | --- | --- | --- | --- | --- | --- | --- | --- |
| Numbers of participants^a^ | | 99 | | | 30 | | | 23 | |
| Time point | Experiencing moderate to severe pain, n/N(%) | | 95% *CI* | Experiencing moderate to severe pain, n/N(%) | | 95% *CI* | Experiencing moderate to severe pain, n/N(%) | | 95% *CI* |
| Predose | 51/99(51.5) | | (41.7, 61.4) | 14/30(46.7) | | (28.8, 64.5) | 13/23(56.5) | | (36.3, 76.8) |
| 0.5 h postdose | 27/98 (27.6) | | (18.7, 36.4) | 7/29 (24.1) | | (8.6, 39.7) | 7/23 (30.4) | | (11.6, 49.2) |
| 1 h postdose | 24/97 (24.7) | | (16.2, 33.3) | 8/30 (26.7) | | (10.8, 42.5) | 6/23 (26.1) | | (8.1, 44.0) |
| 2 h postdose | 24/97 (24.7) | | (16.2, 33.3) | 10/29 (34.5) | | (17.2, 51.8) | 4/23 (17.4) | | (1.9, 32.9) |
| 24 h postdose | 10/96 (10.4) | | (4.3, 16.5) | 4/28 (14.3) | | (1.3, 27.2) | 4/23 (17.4) | | (1.9, 32.9) |
| 48 h postdose | 9/89 (10.1) | | (3.8, 16.4) | 2/27 (7.4) | | (-2.5, 17.3) | 2/23 (8.7) | | (-2.8, 20.2) |

**Abbreviations:** ^a^ Number of participants included in the analysis. FP, full population; PNR, prior nonresponder; RE, rimegepant and eptinezuma

**Appendix 2 Sensitivity analysis in the group with VHFM days**

**Supplementary Table 2. Demographic and baseline characteristics**

| **Demographic** | **VHFM(n=41)** |
| --- | --- |
| Age, mean (SD), y | 41.0 (11.0) |
| Female, n (%) | 28 (68.3) |
| BMI, mean (SD), kg/m^2^ | 22.8 (3.5) |
| Age at migraine onset, mean (SD), y | 10.7 (9.0) |
| Number of accompanying symptoms, n (%) |  |
| 0 | 0 (0.0) |
| 1 | 5 (12.2) |
| 2 | 6 (14.6) |
| ≥3 | 30 (73.2) |
| Accompanying symptoms, n (%) |  |
| Dizziness | 16 (39.0) |
| Nausea | 28 (68.3) |
| Vomiting | 26 (63.4) |
| Phonophobia | 30 (73.2) |
| Photophobia | 25 (61.0) |
| Others | 23 (56.1) |
| Family history of migraine, n (%) | 24 (58.5) |
| Menstrual related migraine, n (%)^a^ | 16 (57.1) |
| Primary migraine type, n (%) |  |
| Migraine without aura | 28 (68.3) |
| Migraine with aura | 13 (31.7) |
| Unknown^b^ | 0 (0.0) |
| MMDs in the past month, median (IQR), d | 21.0 (16.0-28.0) |
| Drug treatments in past month, n (%) |  |
| Participants on acute treatment | 34 (82.9) |
| Participants on preventive treatment | 16 (39.0) |
| Participants on acute and preventive treatments | 11 (26.8) |
| AEs in the past month, n (%) | 21 (51.2) |
| Nonresponse in the past month, n (%) |  |
| Nonresponse to any acute treatment | 7 (17.1) |
| Nonresponse to any preventive treatment | 10 (24.4) |
| Nonresponse to any acute or preventive treatment | 14 (34.1) |
| HIT-6 score, mean (SD)^c^ | 67.4 (6.1) |
| Severe impact (60-78), n (%)^c^ | 36 (90.0) |
| Substantial impact (56-59), n (%) ^c^ | 4 (10.0) |
| Some impact (50-55), n (%)^c^ | 0 (0.0) |
| Little or no impact (36-49), n (%)^c^ | 0 (0.0) |
| MSQ score, mean (SD)^c^ |  |
| Role restrictive | 35.9 (20.3) |
| Role preventive | 43.9 (22.7) |
| Emotional function | 51.7 (28.0) |

**Abbreviations:** No, number; SD, standard deviation; y, years; BMI, body mass index; MMDs, monthly migraine days; NSAIDs, nonsteroidal anti-inflammatory drugs; AEs, adverse events; HIT-6, Headache Impact Test-6; VHFM, very high-frequency migraine

^a^ Measured only in women.

^b^ Participants reported that they were not sure about their aura symptoms during previous migraine attacks

^c^ Participants number=40 for VHFM days.


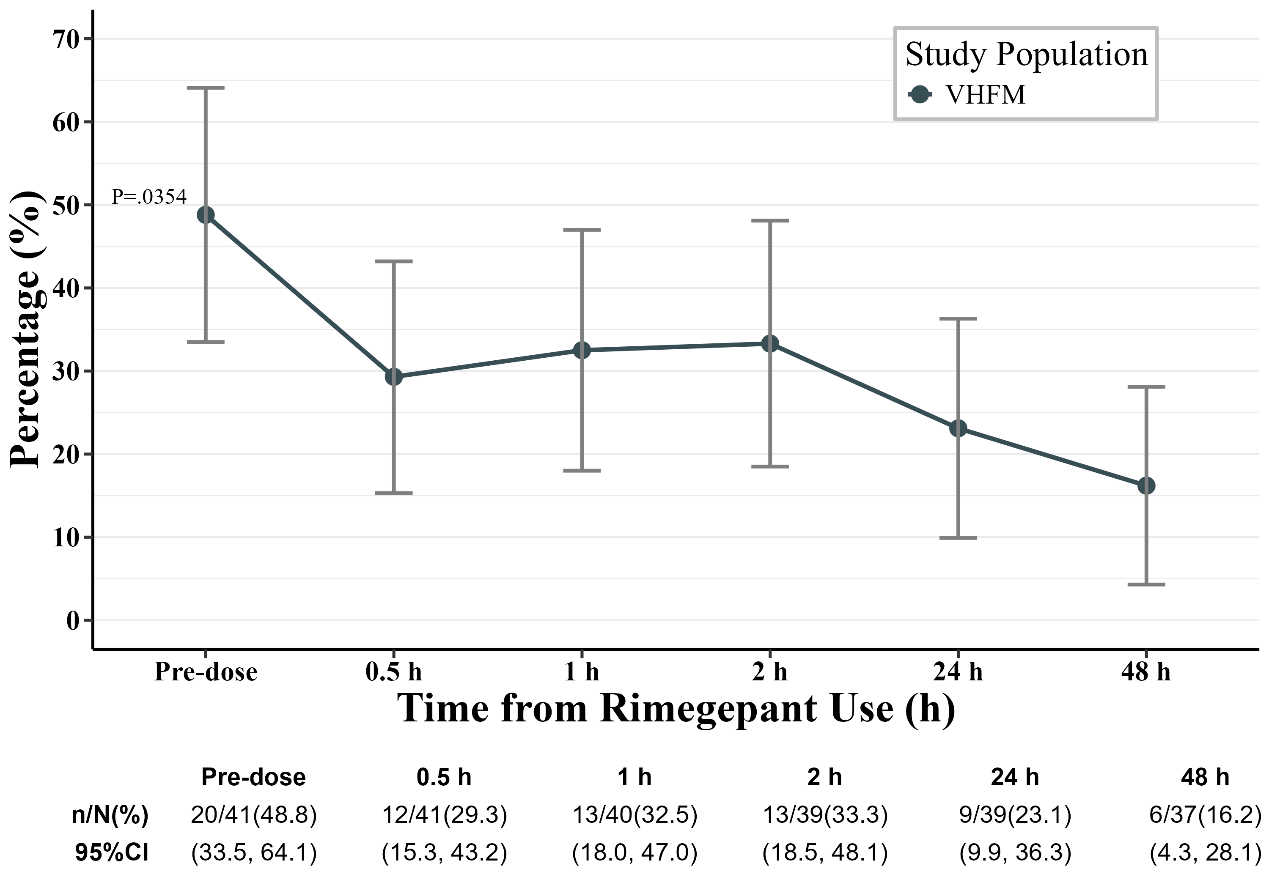


**Supplementary Figure 1.** Percentage of participants who experienced moderate to severe pain at different time points in the study population

**Abbreviations:** VHFM, very high-frequency migraine
